# Corn Snakes Show Consistent Sarcomere Length Ranges Across Muscle Groups and Ontogeny

**DOI:** 10.1093/iob/obac040

**Published:** 2022-09-08

**Authors:** Derek J Jurestovsky, Jessica L Tingle, Henry C Astley

**Affiliations:** Biomechanics Laboratory, Department of Kinesiology, Pennsylvania State University, University Park, PA 16802, USA; Department of Biology, University of Akron, 302 E. Buchtel Avenue, Akron, OH 44325, USA; Department of Biology, University of Akron, 302 E. Buchtel Avenue, Akron, OH 44325, USA; Department of Biology, University of Akron, 302 E. Buchtel Avenue, Akron, OH 44325, USA

## Abstract

The force-generating capacity of muscle depends upon many factors including the actin-myosin filament overlap due to the relative length of the sarcomere. Consequently, the force output of a muscle may vary throughout its range of motion, and the body posture allowing maximum force generation may differ even in otherwise similar species. We hypothesized that corn snakes would show an ontogenetic shift in sarcomere length range from being centered on the plateau of the length-tension curve in small individuals to being on the descending limb in adults. Sarcomere lengths across the plateau would be advantageous for locomotion, while the descending limb would be advantageous for constriction due to the increase in force as the coil tightens around the prey. To test this hypothesis, we collected sarcomere lengths from freshly euthanized corn snakes, preserving segments in straight and maximally curved postures, and quantifying sarcomere length via light microscopy. We dissected 7 muscles (spinalis, semispinalis, multifidus, longissimus dorsi, iliocostalis (dorsal and ventral), and levator costae) in an ontogenetic series of corn snakes (mass = 80–335 g) at multiple regions along the body (anterior, middle, and posterior). Our data shows all of the muscles analyzed are on the descending limb of the length-tension curve at rest across all masses, regions, and muscles analyzed, with muscles shortening onto or past the plateau when flexed. While these results are consistent with being advantageous for constriction at all sizes, there could also be unknown benefits of this sarcomere arrangement for locomotion or striking.

## Introduction

Sarcomeres are the fundamental component of skeletal muscle and despite their microscopic size, they can have dramatic consequences on whole animal performance (Bennett 1985; Taylor 2000; Gidmark et al. 2013). Sarcomeres are composed of thick and thin filaments, myosin and actin, respectively, that slide past each other and generate force (Huxley 1963). Furthermore, the sarcomere's position on the length-tension curve has a direct effect on the amount of force it can generate due to overlap and interactions between the filaments, with the maximum being across the plateau region (i.e., 2.2–2.1 μm in vertebrates) (Gordon et al. 1966). Within vertebrates, sarcomeres are typically found to operate over lengths from 3.6–1.2 μm (Burkholder and Lieber 2001).

Initial position on the length-tension curve and subsequent length change has important implications for animal biomechanics in a variety of contexts. In fish, sarcomere lengths have been shown to influence bite force at different gapes relevant to prey size (Gidmark et al. 2013; Kaczmarek and Gidmark 2020). In frogs, sarcomeres shorten across large length ranges during jumping, but different muscles show different sarcomere lengths in the pre-jump posture. The semimembranosus, a hip extensor, shortens across the plateau for increased force and power production during jumping (Lutz and Rome 1996). In contrast, the plantaris muscle (an ankle extensor with an elastic tendon) shortens from the descending limb onto the plateau to match the increasing resistance while stretching the elastic tendon (Azizi and Roberts 2010; Astley and Roberts 2012). Additionally, sarcomere length has been shown to aid in stability during locomotion via preflexes at timescales too fast for neural feedback due to the rising force on the ascending limb (along with contributions from the force-velocity relationship) (Wagner and Blickhan 1999; Dickinson et al. 2000; Hooper et al. 2016).

Snakes have a complex repeating sequence of muscles along their axial skeleton (Gasc 1974, 1981) which faces multiple demands from several modes of locomotion as well as capturing and restraining prey. A snake could have sarcomeres at the plateau of the length-tension curve when the body is straight to ensure near maximum force and power generation during lateral undulation. Conversely, sarcomeres starting on the descending limb and ending on the plateau would be beneficial for constriction by providing increasing force as the coil of the snake tightens around its prey. Snake sarcomere lengths could also potentially differ between muscles and along the body to correspond to different functions such as force generation and stability. Furthermore, these muscles could also vary along with age/size, with small snakes having sarcomere lengths advantageous for speed to evade predators and adults having sarcomere lengths advantageous for constriction. This ontogenetic shift would prioritize locomotion when predation pressure is highest in neonates and juveniles (Mushinsky and Miller 1993). As the snakes increase in size and predation pressure declines, sarcomere lengths could shift to be advantageous for constriction to prioritize the capture of larger prey (Rush et al. 2014).

We hypothesized that an ontogenetic series of sarcomeres in corn snakes would show a shift from being centered on the plateau of the length-tension curve in small individuals to being on the descending limb in adults. To test this hypothesis, we obtained corn snakes (*Pantherophis guttatus*, Linnaeus, 1766) (*n* = 5) across a wide size range (80–335 g), and measured sarcomere length across sizes, muscles, locations on the body, and posture (straight vs. bent).

## Materials and methods

Five wild-caught corn snakes, *P. guttatus*, were obtained from a commercial provider (snout-vent length, SVL, range 69.5–101.0 cm; mass range 80–335 g). This species was chosen because they are locomotor generalists, constrictors, and wild-caught specimens are readily available. All experiments were approved by University of Akron IACUC.

**Fig. 1:**
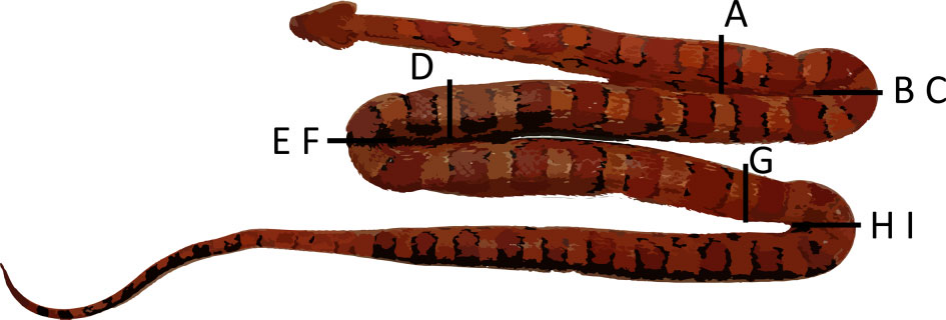
Corn snake preservation posture, showing three maximal bends at 25%, 50%, and 75% SVL. A, D, and G denote regions where straight sarcomere lengths were taken. B–C, E–F, and H–I denote regions where extended and flexed sarcomere lengths were taken.

Snakes were chosen to have an approximately even distribution of log_10_ transformed masses and were either euthanized upon arrival or housed and fed weekly with water ad libitum until reaching an appropriate mass. Corn snakes were euthanized using MS-222 following the two-stage injection protocol in Conroy et al. (2009). After euthanasia, snakes were measured, placed on a corrugated plastic board, and positioned with maximal lateral bending at 25% (anterior), 50% (middle), and 75% (posterior) SVL and a straight posture in all other regions (Fig. 1). We did not wait for rigor mortis to set in, as it would take long enough that the snake would begin to decompose over time. Dissection pins adjacent to, but not piercing, the body were used to hold the posture, and small incisions were made into the skin and formalin was injected into the body cavity to ensure thorough fixation of the musculature. After fixation, we dissected the five largest epaxial muscles, the spinalis, semispinalis, multifidus, longissimus dorsi, and iliocostalis (dorsal and ventral), and one hypaxial muscle, the levator costae (Jayne 1985; Penning 2018). Once the muscles were dissected, they were placed into 15% nitric acid for 3–5 days to loosen the connective tissue and allow easy separation of the muscle fibers. The muscle fibers were photographed using a MU1000 microscope camera connected to a FMA050 adaptor (AMScope, Irvine, CA, USA) under a microscope (40x magnification) and sarcomere lengths were measured using FIJI (Schindelin et al. 2012); ImageJ 1.8.0_66 64 bit, (Wayne Rasband, NIH, Bethesda, MD, USA). A minimum of 20 sarcomeres in series were measured per muscle fiber with 7 muscle fibers measured per muscle and posture. A calibration slide with 0.01 mm spacing was used to convert pixels to mm. In bent postures, measurements were repeated bilaterally to quantify flexed and extended lengths.

For our statistical analysis, we ran an ANCOVA with Type III sums of squares to determine the effects of snake mass (which was a continuous covariate), muscle (spinalis, semispinalis, multifidus, longissimus dorsi, dorsal iliocostalis, ventral iliocostalis, and levator costae), region (anterior, middle, posterior), and posture (straight, flexed, extended) on sarcomere length (package *car*, R 3.6.0 on Windows 10; Fox and Weisberg 2018; R Core Team 2019). Because we measured several sarcomeres for each combination of muscle, region, and posture, we averaged the sarcomere length for each combination and used mean sarcomere length as our response variable. We initially included all two-way interactions in the model, but most were not significant, so we removed non-significant interactions to create our final model. The final model had an adjusted R-squared of 0.904.

## Results

The average length of all sarcomeres measured was 2.50 μm. Sarcomeres in the flexed postures had an average length of 1.79 μm, sarcomeres in the straight postures had an average length of 2.46 μm, and sarcomeres in the extended postures had an average length of 3.26 μm (Table 1). ANCOVA results are presented in Tables 2 and 3.

**Table 1:** Sarcomeres in the corn snake showing flexed, straight, and extended lengths in μm with mean ± s.d. for all seven muscles. Measurements are combined for all masses and regions (i.e., anterior, middle, posterior).

Muscle	Flexed	Straight	Extended
Spinalis	1.72 ± 0.28	2.46 ± 0.21	3.11 ± 0.29
Semispinalis	1.73 ± 0.28	2.43 ± 0.24	3.05 ± 0.33
Multifidus	1.76 ± 0.20	2.41 ± 0.20	3.25 ± 0.30
Longissimus dorsi	1.76 ± 0.28	2.47 ± 0.31	3.45 ± 0.22
Iliocostalis dorsal	1.84 ± 0.29	2.50 ± 0.21	3.36 ± 0.25
Iliocostalis ventral	1.87 ± 0.29	2.50 ± 0.18	3.32 ± 0.24
Levator costae	1.88 ± 0.23	2.42 ± 0.22	3.29 ± 0.22

**Table 2:** Results of ANCOVA analysis showing the effects of snake mass, muscle (spinalis, semispinalis, multifidus, longissimus dorsi, dorsal iliocostalis, ventral iliocostalis, and levator costae), region (anterior, middle, posterior), and posture (straight, flexed, extended) on sarcomere length. We initially included all two-way interactions in the model, but most were not significant, so we removed non-significant interactions in our final model. The final model has an adjusted R-squared of 0.904.

Variable	Sum Sq.	Df	F Value	*P*-value
Intercept	32.369	1	828.3361	<<0.0001
Muscle	0.42	6	1.793	0.1
Region	0.181	2	2.315	0.1
Posture	15.227	2	194.8357	<<0.0001
Mass	0.016	1	0.4135	0.521
Muscle*Posture	1.019	12	2.1737	0.013
Muscle*Mass	0.588	6	2.5062	0.022
Residuals	11.137	285	–	–

**Table 3:** Estimated partial coefficients from the ANCOVA. Note that for categorical variables, one category is used as the base category against which other categories are compared. For example, for posture, the flexed and extended categories are compared to the straight category, so straight is not listed in the table. Middle is the base category for region, and levator costae is the base category for muscle (because it is the muscle whose average sarcomere length is closest to the overall average sarcomere length).

		Partial coefficient	Std. error	t-value	*P*-value
Intercept	–	2.408	0.084	28.781	<<0.0001
Muscle	Iliocostalis (dorsal)	0.308	0.116	2.649	0.009
	Iliocostalis (ventral)	0.041	0.116	0.351	0.726
	Longissimus dorsi	0.255	0.116	2.198	0.029
	Multifidus	0.165	0.116	1.418	0.157
	Semispinalis	0.122	0.116	1.049	0.295
	Spinalis	0.111	0.116	0.956	0.340
Region	Anterior	–0.026	0.027	–0.959	0.338
	Posterior	–0.059	0.027	–2.148	0.033
Posture	Flexed	–0.546	0.072	–7.560	<<0.0001
	Extended	0.867	0.072	12.012	<<0.0001
Mass	–	0.000	0.000	0.643	0.521
Interactions	Iliocostalis (dorsal) * flexed	–0.118	0.102	–1.157	0.248
	Iliocostalis (ventral) * flexed	–0.086	0.102	–0.842	0.401
	Longissimus dorsi * flexed	–0.177	0.102	–1.729	0.085
	Multifidus * flexed	–0.102	0.102	–1.002	0.317
	Semispinalis * flexed	–0.153	0.102	–1.498	0.135
	Spinalis * flexed	–0.199	0.102	–1.949	0.052
	Iliocostalis (dorsal) * extended	–0.011	0.102	–0.108	0.914
	Iliocostalis (ventral) * extended	–0.050	0.102	–0.487	0.627
	Longissimus dorsi * extended	0.104	0.102	1.014	0.311
	Multifidus * extended	–0.028	0.102	–0.277	0.782
	Semispinalis * extended	–0.244	0.102	–2.388	0.018
	Spinalis * extended	–0.220	0.102	–2.160	0.032
	Iliocostalis (dorsal) * mass	–0.001	0.000	–2.490	0.013
	Iliocostalis (ventral) * mass	0.000	0.000	0.416	0.678
	Longissimus dorsi * mass	–0.001	0.000	–2.215	0.028
	Multifidus * mass	–0.001	0.000	–1.967	0.050
	Semispinalis * mass	–0.001	0.000	–1.278	0.202
	Spinalis * mass	0.000	0.000	–0.776	0.438

Mass did not significantly predict sarcomere length (F_1,285_ = 0.414, *P* = 0.521), and sarcomere length did not differ significantly by body region (anterior vs. middle vs. posterior; F_2,285_ = 2.315, *P* = 0.101) or muscle (F_6,285_ = 1.793, *P* = 0.100) (Table 2). Body posture did significantly predict sarcomere length: sarcomeres in flexed regions were 0.546 μm shorter (partial coefficient, Table 3) than those in straight regions (a decrease of ∼22% the average sarcomere length; 95% confidence interval 0.404–0.688 μm), whereas sarcomeres in extended regions were 0.867 μm longer (partial coefficient, Table 3) than those in straight regions (an increase of ∼35% the average sarcomere length; 95% confidence interval 0.725–1.009 μm) (F_2,285_ = 194.836, *P* << 0.0001). Two significant two-way interactions included muscle*posture and muscle*mass (F_12,285_ = 2.174, *P* = 0.013 and F_6,285_ = 2.506, *P* = 0.022, respectively).

## Discussion

**Fig. 2:**
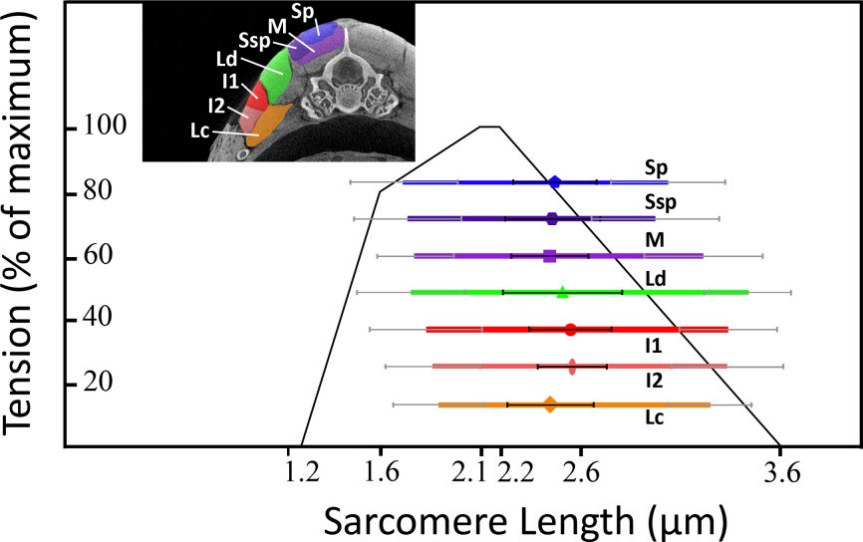
A length-tension curve showing all snake muscles analyzed in this study from five corn snakes. Shapes are placed at straight lengths and the ends of the color bars represent extended and flexed sarcomere lengths, respectively. Smaller gray and black bars represent standard deviation for each element. Inset in the top left shows a μCT scan of a corn snake body segment stained with PMA with relevant muscles highlighted and labeled (Sp, blue, pentagon-spinalis; Ssp, dark purple, hexagon-semispinalis; M, light purple, square-multifidus; Ld, green, triangle-longissimus dorsi; I1, red, circle-dorsal iliocostalis; I2, light red, oval-ventral iliocostalis; and Lc, orange, diamond-levator costae).

Corn snake sarcomeres had large differences between extended, straight, and flexed lengths (Fig. 2, Table 1). Different muscles, body sizes, and regions on the body showed only minor differences in sarcomere lengths and thus were combined in Fig. 2. Straight and extended lengths of sarcomeres for every muscle were on the descending limb of the length-tension curve. Flexed lengths of sarcomeres for most muscles were on the ascending limb, with only a few exceptions on the plateau of the length-tension curve (Fig. 2). It should be noted that these sarcomere values are based on predictions from the standard sliding filament model for vertebrates and does not include possible variation in curve shape based on measured performance or the passive curve of muscles elastic properties (Gordon et al. 1966).

We found the sarcomere positions on the length-tension curve (Fig. 2) are consistent with being advantageous for a snake that constricts its prey. As the muscles contract and the body bends, they generate higher forces and make it more difficult for the prey to escape the constricting coils. However, while this is consistent with constriction performance, we cannot say it evolved for that reason based on only a single species (Gould and Lewontin 1979; Felsenstein 1985; Garland and Adolph 1994). Contrary to our hypothesis, sarcomere length did not change with size, so the sarcomeres are not shifting during ontogeny to optimize from one function (i.e., locomotion) to another (i.e., constriction). This result could indicate that the relative necessity of constriction versus fast locomotion does not change with body size in corn snakes, and that selection therefore does not favor an ontogenetic change in sarcomere length. It is also possible that selection is acting on other aspects of performance, potentially related to locomotion or other behaviors such as striking. Additionally, non-selective factors could be at play, such as phylogenetic inertia and/or developmental constraints.

It was not surprising for sarcomeres to be significantly longer in extended muscles and significantly shorter in flexed muscles. However, it was surprising that the estimated coefficients for the flexed and extended postures had different magnitudes (with non-overlapping confidence intervals), indicating that the extended and flexed lengths are different distances from the length of sarcomeres at the muscles’ resting positions. One possibility is that muscle shortening is constrained by lateral muscle bulging of adjacent muscle bellies. Muscles are constant volume hydrostats, and as such they laterally bulge as they shorten, which can put pressure on adjacent muscles and structures in the body limiting the range of geometries that a muscle fiber can occupy (Brainerd and Azizi 2005). However, it should be noted that the difference in the magnitude of estimated coefficients could be an artifact of our preservation methods, such as not allowing the snakes to go into rigor mortis before placing them into formalin (to prevent decomposition).

Having muscles on the descending limb of the length-tension curve could have complex effects on locomotion. For example, if a snake is perturbed in the direction of an active contraction, it will increase the contraction force further and assist the motion. If the perturbation is in the opposite direction of the contracting segment, the body would stretch as the muscle would lose force due to the effects of the length-tension curve (partially counteracted by the force increase due to eccentric muscle loading). This could harm the snakes’ ability to maintain its posture during locomotion, but could also help the snake passively conform to the substrate. However, our results only show the change in force due to length-tension relationships in snake muscle, and during active locomotion multiple other factors influence force production, including the force-velocity relationship and activation-deactivation dynamics (Josephson 1985). Finally, while being on the descending limb is a risk for sarcomeres popping (Morgan 1990), this could be mitigated by tendons stretching.

This is the first study to analyze sarcomere lengths in the locomotory muscles in snakes. Accordingly, it is unclear if this pattern is conserved among snakes or if variation exists based on a multitude of factors including phylogenetic history, constrictors versus non-constrictors, habitat type (e.g., terrestrial, arboreal, aquatic, and burrowing), and taxa that have developed venom. However, sarcomere lengths have been reported in a variety of other taxa including frogs, mice, rats, rabbits, fish, cats, birds, and humans (Burkholder and Lieber 2001). The majority of these taxa have highly variable sarcomere lengths (see Burkholder and Lieber 2001, and references therein) but two exceptions included bird (pectoralis, patagialis, and surpracoracoideus) and fish (red and white axial muscle) sarcomeres that were both positioned almost entirely on the plateau and the ascending limb (Cutts 1986; Ashmore et al. 1988; Mathieu-Costello 1991; Rome and Sosnicki 1991; Lieber et al. 1992). Both of these groups operate in dynamic habitats that prioritize power production that would benefit from sarcomeres with short excursions near the plateau of the length-tension curve. These restricted sarcomere lengths are unsurprising in these habitats. For birds, power production is important to produce fast wing beats to maintain flight, whereas fish benefit from the plateau of the length-tension curve by activating fewer motor units and the associated energetic savings (Cutts 1986; Rome and Sosnicki 1991). However, it is relatively unclear why corn snake sarcomeres are highly consistent across mass/age, region of the body, and even different muscles. More generally, it is unclear what factors govern the ranges of sarcomere lengths a muscle operates over. Muscles perform a wide variety of tasks from force production to stabilization (Dickinson et al. 2000) and one muscle can perform multiple functions, depending upon stimulation (Full et al. 1998). Thus, functional inferences based on sarcomere length should be regarded tentatively.

The consistent sarcomere length range across postures in a variety of snake muscles, despite highly variable distances from the vertebrae, is puzzling. Theoretically, muscles close to the vertebral midline, such as the spinalis and semispinalis, should have a shorter muscular excursion compared to muscles further away from it, such as the iliocostalis (Full et al. 1998) (see inset in Fig. 2). However, we found a surprising consistency in muscular excursions regardless of distance from the center of rotation. This is unlikely to be a result of mistakes in preservation or other methodological errors due to the high consistency regardless of mass, muscle, region of the body, or tendon length (because muscles with short and long tendons still showed consistent values). One possible explanation for these values could be muscle and tendon length ratios. For example, the iliocostalis is predicted to undergo large length changes due to its distance from the center of rotation, and since it has relatively long muscle fibers with short tendon lengths (Jayne 1988), the relative length change of the muscle fiber will closely match that of the whole muscle-tendon unit (Ruben 1977; Lawing et al. 2012; Astley 2020). In contrast, the semispinalis-spinalis is located close to the midline of the snake and thus expected to undergo only modest length change during lateral bending, but because the anterior tendon comprises over 70% of the length of the muscle-tendon unit (Jayne 1982), the relative length change of the muscle fiber would be correspondingly amplified (Ruben 1977; Lawing et al. 2012; Astley 2020). However, future work investigating snake muscular lever arms will explore this quantitatively. Furthermore, if longer tendons display elastic behavior, the muscle may be able to shorten while the tendon stretches, decoupling muscle length change from body motion (Astley and Roberts 2012). This decoupling and the consequent storage of elastic potential energy would substantially increase the complexity of muscle-tendon unit behavior, potentially allowing for energy savings or power amplification (Roberts and Azizi 2011). However, the stiffness of snake tendons has never been experimentally tested, nor has *in vivo* muscle length change been quantified in snakes, so this possibility must remain speculative until future testing.

## Supplementary Material

obac040_Supplemental_File
